# 

**Published:** 1894-03

**Authors:** 


### CONTENTS—MARCH, 1894.—VOL. IX., NO. 9.

ORIGINAL COMMUNICATIONS—

Cysts of the Spermatic Cord and Testicle. Prof. J. E. Thompson, M. D., Galveston. . . . . 433
Ostitis. Matthew M. Smith, M. D., Austin . . . . . 437
Traumatism as a Factor in Ovarian Pathology. James Kennedy, M. D., Ph. G., Galveston. . . . . 440
Treatment of Uterine Febroids. A. H. Goelet, M. D., . . . . . 444
Do Newspapers Foster Abortion? H. Byder-Taylor, San Antonio . . 446

ABSTRACTS OF CURRENT MEDICAL LITERATURE—

Department of Therapeutics. Edited by Prof. D. Cerna, M. D., Galveston . . . . . 448

SOCIETY NOTES—

Texas State Medical Association . . . . . 452
The Annual Meeting . . . . . 453

EDITORIAL—

An Improved Method of Brain-Localization in Epilepsy . . . . . 455
A Literary Bombshell . . . . . 459
A Mare's Nest . . . . . 461
The New Antipyretic . . . . . 462
The Two Bills in Congress . . . . . 464
Indecent Publications in Newspapers . . . . . 466

MEDICAL NEWS AND MISCELLANY—

Numerous News Items . . . . . 468

DENTAL DEPARTMENT—

"Pyorrhœa Alveolaris" . . . . . 470

SELECTIONS—

The New Antipyretic, 472; A Bill to Establish a Department of Public Health, 474: Puerperal Sepsis; its Prophylaxis and Treatment, 477; The Opium Disease in Infants and Children, 478 . . . 472–478

BOOK NOTICES— . . . . . 478

PUBLISHERS' NOTES . . . . . 484

